# Supplementary material for: Saponin-Rich Extracts and Their Acid Hydrolysates Differentially Target Colorectal Cancer Metabolism in the Frame of Precision Nutrition
Source: Cancers (Basel). 2020 Nov 17;12(11):3399. doi: 10.3390/cancers12113399 (PMC7698026; doi:10.3390/cancers12113399)
Supplement: Supplementary file 1 [file cancers-12-03399-s001.pdf]

cancers

Supplementary Materials: Saponin-Rich Extracts and their Acid Hydrolysates Differentially Target Colorectal Cancer Metabolism in the Frame of Precision Nutrition

Marta Gómez de Cedrón, Joaquín Navarro del Hierro, Marina Reguero, Sonia Wagner, Adrián Bouzas , Adriana Quijada-Freire, Guillermo Reglero, Diana Martín and Ana Ramírez de Molina

List of oligos and Taqman probes used in this study.

SREBF1 F-CCGCCGCGCCTTGAC; R-AGCATAGGGTGGGTCAAATAGG

FASN F-TATGAAGCCATCGTGGACGG; R-GAAGAAGGAGAGCCGGTTGG

SCD-1 F-TGCCCACCACAAGTTTTCAG; R-CATCAGCAAGCCAGGTTTGT

HMGCR F-TGATTGACCTTTCCAGAGCAAG; R-CTAAAATTGCCATTCCACGAGC

APOA1 F-CCCTGGGATCGAGTGAAGGA; R-CTGGGACACATAGTCTCTGCC

ABCA1 F-ACCCACCCTATGAACAACATGA; R-GAGTCGGGTAACGGAAACAGG

FABP1 F-GTGTCGGAAATCGTGCAGAA; R-AGCTCACATTCCTCCCCCA

LDLR F-CAGATATCATCAACGAAGC; R-CCTCTCACACCAGTTCACTCC

ACSL1 F-TTGGGAAGGATTCTGGTCTG; R-TGCCATTTCCTCTG GCTTT

ACSL4 F-GGCACAACAGAAAGGGGTAG; R-GGTTCCTCAGCTCCTTCCTT

B2M F-GATGAGTATGCCTGCCTGCCGTGT; R-TGCGGCATCTTCAAACCTCC

Taqman probes: *CHKA- Hs00957878_m1; BMP2- Hs00154192_m1; NEF2L2- Hs00232352_m1; JAK1-Hs01026983_m1; TYMS - Hs00426591_m1; TK1 - Hs01062125_m1*

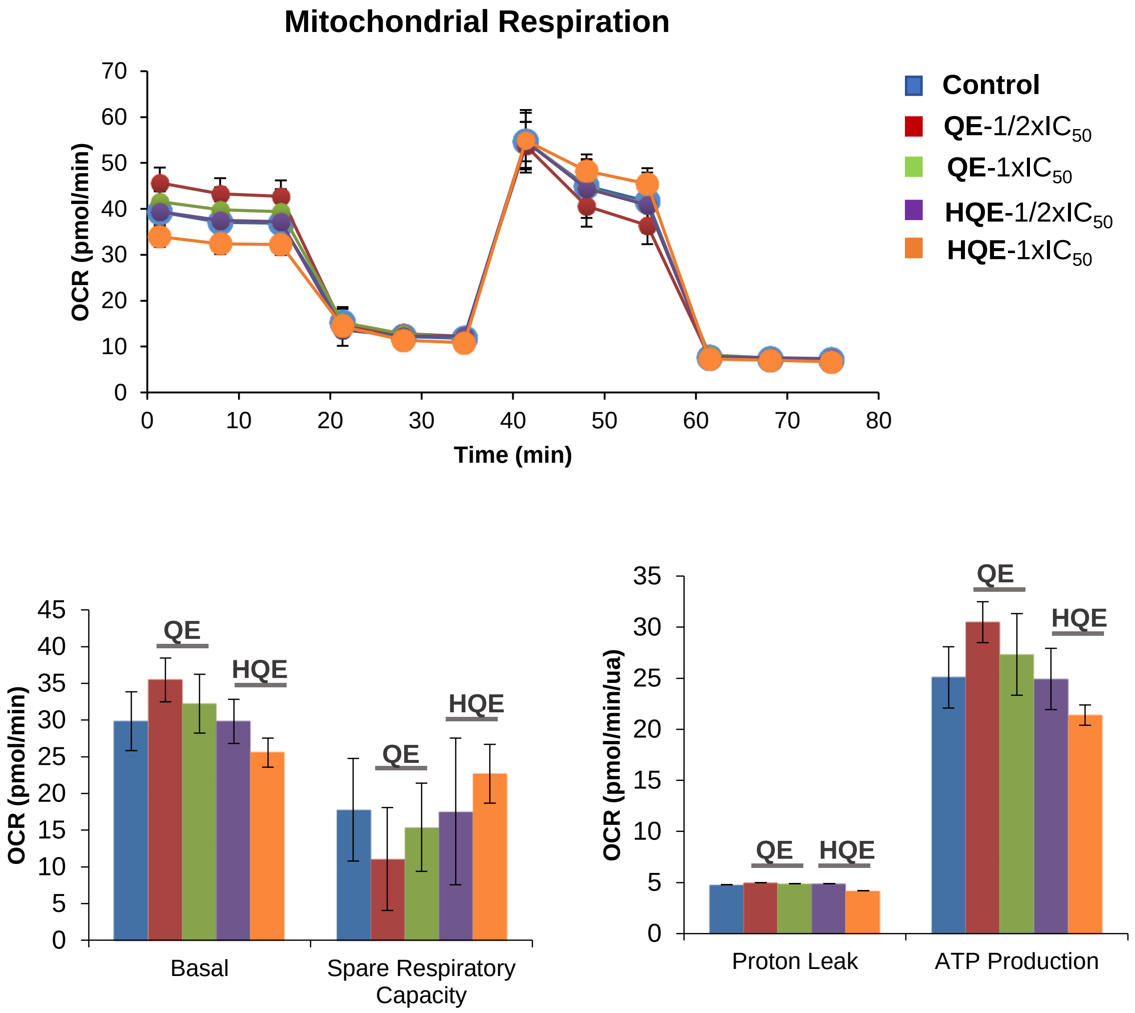

Figure S1. QE and HQE extracts do not inhibit mitochondrial oxidative phosphorylation of CRC cell lines. MitoStress profile and quantification of basal OCR, Maximal Respiration Rate, ATP, Spare respiratory capacity and Proton Leak of DLD1 CRC cells pretreated with Q and Q-Hy (at the indicated doses).

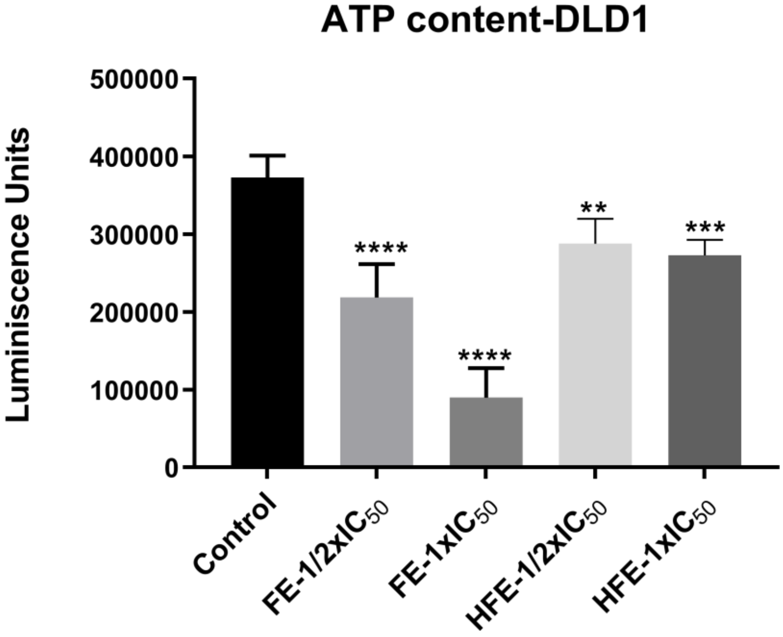

Figure S2. Quantification of ATP intracellular levels of CRC cells pretreated with FE and HFE at the indicated doses. Asterisks indicate statistically significant differences ($p < 0.05$ (*); $p < 0.01$ (**); $p < 0.005$ (***); $p < 0.001$ (****)) relative to the control non-treated cells (3-4 replicates, 3 independent experiments).

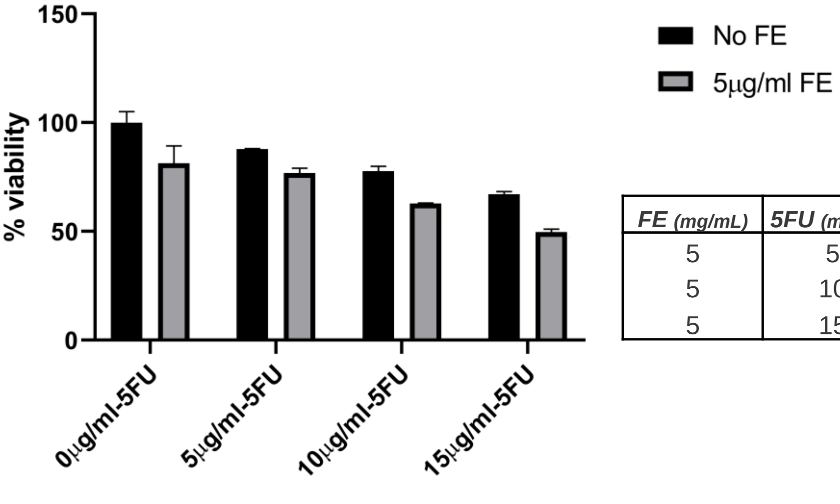

FE (mg/mL)	*5FU (mg/mL)*	*Fa*	*CI*
5	5	0.231785	1.083
5	10	0.37166	1.001
5	15	0.50305	***0.880***

Figure S3. FE synergizes with 5-FU treatment. Quantification of the effects on cell viability of FE extract in combination with the antitumoral drug 5-fluororacil (5-FU) on CRC cells. According to the Chou–Talalay method, the combination of FE with 5-FU resulted in a synergistic effect displaying a combinatory index (CI) value < 1 in the combination FE (constant dose of 5 ug/mL, pre-treatment 4h) and 5-FU (15 µg/mL).

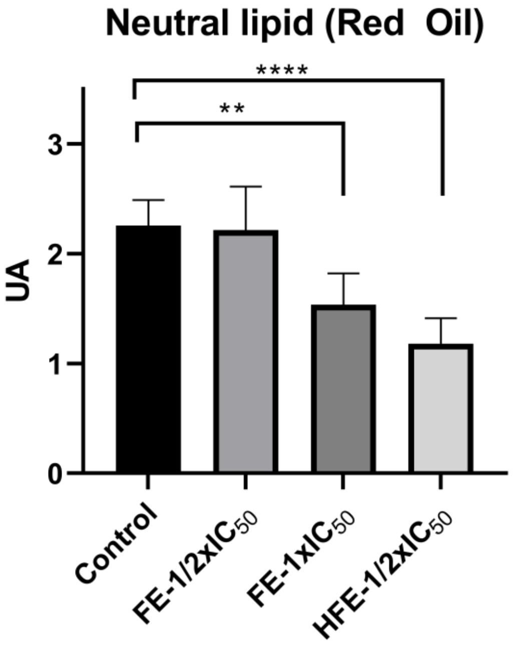

Figure S4. Quantification of intracellular neutral lipid content by Red Oil O staining of CRC cells pretreated for 48 h with HFE compared to control non-treated cells. Asterisks indicate statistically significant differences ($p < 0.05$ (*); $p < 0.01$ (**); $p < 0.005$ (***); $p < 0.001$ (****)) relative to the control non-treated cells (3-4 replicates, 3 independent experiments).
